# A Systems Biology Approach Identifies Molecular Networks Defining Skeletal Muscle Abnormalities in Chronic Obstructive Pulmonary Disease

**DOI:** 10.1371/journal.pcbi.1002129

**Published:** 2011-09-01

**Authors:** Nil Turan, Susana Kalko, Anna Stincone, Kim Clarke, Ayesha Sabah, Katherine Howlett, S. John Curnow, Diego A. Rodriguez, Marta Cascante, Laura O'Neill, Stuart Egginton, Josep Roca, Francesco Falciani

**Affiliations:** 1 School of Biosciences, University of Birmingham, Birmingham, United Kingdom; 2 Hospital Clínic, Institut d'Investigacions Biomèdiques August Pi i Sunyer (IDIBAPS), (CIBERES), University of Barcelona, Barcelona, Spain; 3 Institute of Biomedical Research, School of Immunity and Infection, College of Medical and Dental Sciences, University of Birmingham, Birmingham, United Kingdom; 4 Department of Biochemistry and Molecular Biology, Biology Faculty, Universitat de Barcelona, Biomedicine Institute from Universitat de Barcelona (IBUB), Barcelona, Catalonia, Spain; University of Tokyo, Japan

## Abstract

Chronic Obstructive Pulmonary Disease (COPD) is an inflammatory process of the lung inducing persistent airflow limitation. Extensive systemic effects, such as skeletal muscle dysfunction, often characterize these patients and severely limit life expectancy. Despite considerable research efforts, the molecular basis of muscle degeneration in COPD is still a matter of intense debate. In this study, we have applied a network biology approach to model the relationship between muscle molecular and physiological response to training and systemic inflammatory mediators. Our model shows that failure to co-ordinately activate expression of several tissue remodelling and bioenergetics pathways is a specific landmark of COPD diseased muscles. Our findings also suggest that this phenomenon may be linked to an abnormal expression of a number of histone modifiers, which we discovered correlate with oxygen utilization. These observations raised the interesting possibility that cell hypoxia may be a key factor driving skeletal muscle degeneration in COPD patients.

## Author Summary

Chronic Obstructive Pulmonary Disease (COPD) is a major life threatening disease of the lungs, characterized by airflow limitation and chronic inflammation. Progressive reduction of the body muscle mass is a condition linked to COPD that significantly decreases quality of life and survival. Physical exercise has been proposed as a therapeutic option but its utility is still a matter of debate. The mechanisms underlying muscle wasting are also still largely unknown. The results presented in this paper show that diseased muscles are largely unable to coordinate the expression of muscle remodelling and bioenergetics pathways and that the cause of this phenomena may be tissue hypoxia. These findings contrast with current hypotheses based on the role of chronic inflammation and show that a mechanism based on an oxygen driven, epigenetic control of these two important functions may be an important disease mechanism.

## Introduction

Chronic Obstructive Pulmonary Disease (COPD) is an inflammatory process of the lung that generates progressive and largely poorly reversible airflow limitation [1], [2]. COPD represents a high burden on healthcare systems worldwide, since it is the fourth cause of death and its prevalence is expected to increase in forthcoming years [3].

The disease is primarily caused by the interplay between inhaled irritants, most frequently tobacco smoking but also environmental pollutants, and influenced by genetic susceptibility [4]. In these patients, the disease results in shortness of breath and contributes to limitation of exercise tolerance, leading to a decrease in daily physical activities [5], [6]. The latter has a significant deleterious impact on both clinical outcomes and prognosis [7], [8], [9]. Rehabilitation programs including skeletal muscle training and promotion of active lifestyles are recommended by all international clinical guidelines [4] as pivotal elements in the therapeutic strategies for COPD, but they are insufficiently deployed. One of the reasons for this is that in a significant percentage of patients, skeletal muscle dysfunction and muscle wasting are hallmark systemic effects of COPD [10]. Possibly linked with this, exercise-induced oxidative stress in COPD muscles is well documented and is likely to be an important mechanism driving tissue degeneration [11], [12], [13].

The role of systemic inflammation and myogenesis in skeletal muscle wasting are still a matter of controversy. However, recent studies have shown a reduction in the expression of myogenic genes in COPD muscles [14], [15] and a reduction in the ability to induce their expression in response to training in cachectic COPD patients [16], providing evidence for a deficiency in tissue remodelling. It has been proposed that lack of activation of myogenic pathways may be the result of the over-activation of the NF-kB pathway induced by systemic inflammatory signals generated by the lung [14]. This hypothesis is supported by cell culture and animal experiments, but so far there has been little evidence that this mechanism is clinically relevant [15]. Analysing a panel of muscle biopsies from normal and COPD individuals these authors showed that COPD muscles may be unable to activate NF-kB targets in response to physical training. Therefore, the mechanisms leading to skeletal muscle abnormalities in COPD and the relationship between muscle remodelling and oxidative damage are still a matter of intense research, and the extensive literature in this field is unable to support the development of effective predictive/preventive strategies.

The complex interplay of molecular pathways that are potentially involved in regulating muscle functionality makes a systems biology approach a desirable option. Such an approach aims to model the relationship between key molecular and physiological variables in healthy and diseased individuals to derive a testable hypothesis on the disease mechanism.

In this study, we hypothesized that skeletal muscle abnormalities in COPD may be the result of an imbalance in the physiological regulation of normal muscle homeostasis induced by systemic inflammatory mediators and chronic tissue hypoxia. In addition, we assume that the nature of such alteration might be reverse engineered by observing the statistical relationship between variables defining whole body physiology, systemic inflammation and muscle transcriptional state, with particular reference to cell bioenergetics and tissue remodelling functions. We based our analysis on a clinical study representing 12 healthy subjects and 18 age and sex-matched COPD patients, before and after undergoing an 8 weeks training program. In the latter category we included patients with preserved muscle mass (COPD_N_) and patients showing muscle wasting (COPD_L_).

We discovered that COPD muscles are characterized by a lack of correlation in expression of bioenergetic and tissue remodelling pathways, which included genes specifically involved in myogenesis. Our analysis suggests that failure to activate and coordinate these functions in response to training is associated with a general lack of activation of NF-kB targets, including many pro-inflammatory signals (e.g. IL-1β). We also discovered that expression of chromatin modification enzymes, known to control muscle differentiation and energy balance in other biological systems, is abnormal in COPD muscles and correlated with oxygen availability. This finding raises the possibility that an epigenetic mechanism triggered by tissue hypoxia may be the basis of skeletal muscle wasting in COPD.

## Methods

### Ethics statement

All animal work has been conducted according to relevant national and international guidelines and approved by the University of Birmingham, Medical School ethics committee.

### Study groups and design

The 8 weeks training project (TP) was a clinical investigation in which eighteen COPD patients (68±7 yrs, 17 men, FEV_1_ 46±12% predicted and PaO_2_ 75±0.7 mmHg) with a wide spectrum of body mass composition and twelve age-matched healthy sedentary controls (65 yrs, 10 men, FEV_1_ 107±14% predicted and PaO_2_ 93±0.7 mmHg) underwent a protocol of supervised endurance exercise. The inclusion criteria were: 1) diagnosis of COPD according to GOLD criteria, 2) a stable clinical condition using standard treatment with bronchodilators and inhaled corticosteroids, 3) absence of episodes of exacerbation or oral steroid treatment in the previous 4 months, 4) absence of significant co-morbidities. All procedures were performed in the Pulmonary Function Laboratory or the Rehabilitation Unit at the Hospital Clinic-IDIPAPS. The TP included twelve patients with stable COPD and normal fat free mass index (FFMI, 21 Kg/m^2^) (COPD_N_), six COPD patients with low FFMI (16 Kg/m^2^) (COP_L_), and twelve healthy sedentary subjects (FFMI 21 Kg/m^2^). The study was approved by the Ethics Committee of the Hospital Clinic (Barcelona, Spain) and all patients gave written informed consent. Subjects completed all phases of the protocol as well as fully contributing to the sampling regime.

Significant physiological training effects were obtained using a standard supervised interval training program. Table S1 and Figure S1A in Text S1 summarize the characteristics of the study groups and the training-induced effects on physiological variables. Constant-work rate exercise at 70% of pre-training Watts peak (Wpeak) (CardiO_2_ cycle Medical Graphics Corporation, USA) was carried out before and after 8-weeks training with cycloergometer, until pre-training endurance time exhaustion. Measurements before and after training were obtained at isowork-rate and iso-time.

### Measurement of inflammatory mediators in serum

Serum samples obtained at rest before training (Basal-BT) and at rest after the eight weeks training program (Basal-AT) were analysed by Luminex xMAP technology, according to the manufacturer's instructions BIO-RAD [17]. A number of cytokines/growth factors were selected for analysis based on the results of a previous proteomic study carried out in COPD patients and controls [18]. In that study [18] a total of 142 cytokines/growth factors were analysed and 42 of them were differentially expressed in COPD. Our selection includes 30 analytes covering ∼50% of the cytokines/growth factors previously identified [18], which demonstrated association to clinical parameters in the same study. The complete list of proteins measured and the corresponding results of the analysis is shown in Table S2 and Figure S1B in Text S1.

### Expression profiling of muscle biopsies

Skeletal muscle transcriptomics was performed on open biopsies from the *m. vastus lateralis* (quadriceps). In all participants these were obtained at rest, before and after training. RNA was isolated using RNeasy extraction kits (QUIAGEN, USA) according to the manufacturer instructions. Microarray gene expression analysis employing Affymetrix ® GeneChip technology was performed using Human U133 Plus2 Gene Chips according to the manufacturer's suggested protocols. Data were subsequently subject to quality control to assess integrity of the RNA using an Agilent Bioanalyzer (Agilent). These datasets were normalised using the R library gcrma, which converts CEL files using a robust multi-array average (RMA) expression measure with the help of probe sequences. Data were quantile normalised [19].

### Identification of statistically significant differences in physiology, serum protein and muscle gene expression measurements

In order to identify differential changes in physiological and protein measurements we used two-factor ANOVA with disease and training as factors. Significantly different variables were selected using a threshold of *P*<0.01. Similarly, genes differentially expressed between sedentary and trained subjects in the three populations (healthy, COPD_N_ and COPD_L_ patients) were identified by t-test followed by Benjamimi-Hochberg multiple correction [20] using a false discovery rate (FDR) threshold of *q*<10%.

### Network inference

#### Building a model including mRNA, protein and physiology measurements representing pre and post-training samples in the 8 weeks study

In order to develop molecular networks representing the interaction between systemic and local signals, we have integrated physiology, blood cytokine levels and muscle mRNA expression measurements by standardizing each variable, across all samples to have mean of 0 and standard deviation of 1 using the R package QuantPsyc [21]. After this procedure, measurements, in different units become directly comparable. We engineered a global interaction network as the union of individual network modules, each one of them defined as a cluster of variables linked by a significant correlation to the profile of key biomarkers. Such biomarkers, which can be thought of as network hubs, were chosen to represent biological factors we hypothesized could be important players in the context of the clinical problem. These can be summarized in four distinct groups (for a detailed list see Table S3 in Text S1):

1. Indicators of whole body physiology.
2. Serum protein levels representing systemic inflammatory signals.
3. Muscle mRNA expression profiles of genes encoding for receptors of cytokines and growth factors, known to play a role in muscle physiology.
4. Muscle mRNA expression profiles of genes encoding for enzymes in the glycolysis/gluconeogenesis pathway.

The ability to infer relationships between measured variables from observational data is at the core of this study. For this reason we have chosen ARACNE [22], a validated network inference technique that infers the interaction between pairs of variables using a measure of correlation called mutual information (MI) [22], [23]. Sparse networks are generated by eliminating non-significant connections according to a permutation-based statistical test. Although ARACNE has been used mainly in association with gene expression data it can be applied to infer interactions between any variable for which we have quantitative measurements. ARACNE offers numerous advantages over more traditional measures of correlation, including the ability to spot non-linear correlations, which are very effective in identifying biologically relevant connections [22].

In order to infer networks integrating multi-level measurements from the eight weeks training study we initially used ARACNE to reconstruct the network neighbourhoods of muscle-expressed intermediate metabolism genes, immune system and growth factor receptors genes (measured in muscle biopsies, using microarray technology), systemic cytokines (using Luminex technology, measured in serum), and physiological measures (Table S3 in Text S1). Statistically significant network edges have been selected using a threshold p<10^−7^ and no edges have been eliminated using DPI. Significant connections were identified by applying a *P*-value threshold for significant MI values that ensure less than 5% false positives [24]. Individual networks generated with ARACNE were merged and the resulting network visualised using the software application Cytoscape [25], a force-driven layout where the strength of association in the graph is determined by the value of mutual information associated with the gene-to-gene connection. This layout allows the representation of a network structure where the position of the nodes is determined by the relative strength of connections. Genes that are strongly linked (i.e. by a high MI value) will appear closely associated in the graph, whereas genes with a weak association will be far apart. This procedure was applied to infer all networks presented in this paper. Moreover, gene lists derived from statistical analysis of microarray data and from the analysis of interaction networks have been functionally annotated using a Fisher test as implemented in the web-based application DAVID [26]. A Benjamini-Hochberg FDR correction for multiple testing was applied and a threshold of FDR<0.01 chosen for selecting significant enrichment of specific Gene Ontology (GO) or Kyoto Encyclopedia of Genes and Genomes (KEGG) terms in the different network modules.

#### Individual network models representing healthy and COPD muscles

In order to address whether the transcriptional uncoupling between genes involved in tissue remodelling and bioenergetics was a specific feature of COPD, the procedure described above was also applied to healthy and COPD muscle datasets. In order to increase the resolution, we have integrated our dataset with a similar microarray study recently performed by Radom-Aizik *et al.* [27]. This dataset provided microarray data representing an additional 12 healthy and 12 age-matched COPD patients pre and post endurance training (12 weeks). This dataset was quantile normalized using the R library gcrma and microarray batch effects were corrected using an Empirical Bayes framework available in R [28].

#### Network models representing diabetes and muscle dystrophy muscle biopsies

We have also analyzed two additional datasets, representing muscle biopsies derived from patients affected by diabetes and dystrophy. The first microarray dataset consists of skeletal muscle biopsy samples from 43 age-matched males with different degrees of glucose intolerance and including Type 2 diabetes mellitus (available at: http://expression.gnf.org/) [29]. The dystrophy dataset consist of 84 samples from patients representing several types of muscle dystrophy (Facioscapulohumeral muscular dystrophy samples, Becker muscular dystrophy, Emery Dreifuss muscular dystrophy, from Duchenne muscular dystrophy) (GEO accession: GSE3307) [30].

### Mouse *in vivo* experiments

To assess the effects of pro-inflammatory cytokines on skeletal muscle, recombinant mouse IL-1β (10 mg/ml) was injected in a single dose into the tail vein of C57BL10 mice at a loading of 100 ng/mouse (4 animals per group) and samples taken from lateral gastrocnemius (glycolytic) and soleus (oxidative) muscles 24 h later, with saline-injected animals acting as controls. Samples were stored at −80°C until use. RNA was extracted using an RNeasy RNA extraction kit (QUIAGEN). Microarray expression profiling of four independent biological replicates was performed using full genome oligonucleotide arrays (OPERON) after labelling the mRNA with the Cy-Scribe post labelling kit (Amersham) according to the manufacturer's instructions. Genes differentially expressed were identified by a t-test followed by multiple testing correction (FDR<10%). Significant genes from the mouse experiment were mapped on the human COPD network by converting mouse gene identities into their human homologues using the database Homologene (data was normalised using the same procedures described for the COPD dataset).

### Ingenuity pathway analysis

The gene set represented by those directly connected to growth factor and cytokine receptor components of the network and differentially expressed in response to training in healthy individuals were analyzed using the Ingenuity Pathway Analysis (IPA) application (Palo Alto, http://www.ingenuity.com), a web-based application that enables discovery, visualization, and exploration of biological interaction networks. Once the gene list was uploaded to the application, each gene identifier was mapped to its corresponding gene object in the Ingenuity Pathways Knowledge Base. These genes, called focus genes, were overlaid onto a global molecular network developed from information contained in the Ingenuity Pathways Knowledge Base. Networks of these focus genes were then algorithmically generated based on their connectivity according to the following procedure implemented in the IPA software application. The specificity of connection for each focus gene was calculated by the percentage of its connection to other focus genes. The initiation and the growth of pathways proceed from the gene with the highest specificity of connections. Each network had a maximum of 35 genes for easier interpretation and visual inspection. Pathways of highly interconnected genes were identified by statistical likelihood. Networks with a Score greater than 20 and containing more than 60% of focus genes were selected for biological interpretation. Canonical pathway and functional term enrichment analysis was performed using the IPA tools, and significance for the enrichment of the genes within a particular Canonical Pathway was determined by right- tailed Fisher's exact test with α = 0.01 using the whole database as a reference set.

### Molecular response of mouse quadriceps muscles to chronic hypoxia conditions

In order to assess whether genes correlated to VO2max in the clinical study are indeed modulated in response to hypoxia we have analysed a publicly available microarray dataset representing the transcriptional response of C57Bl/10 mice to 2 weeks of chronic hypoxia and compared those with matching controls kept in normoxic conditions. The dataset was developed by Budak et al. [31], and it is available in the Gene Expression Omnibus database under the accession number GSE9400. The details of the overall design and the dataset can be found in GEO.

For clarity, here we report a concise summary of the experimental design and data generation. Adult male C57Bl/10 mice were divided into two groups, control (room condition) and hypoxic, over a period of 2 weeks. The hypoxic group was gradually exposed to lower levels of hypoxia in a specially designed and hermetically closed hypoxic chamber. A Pegas 4000 MF (Columbus Instruments) gas blending system was used. The oxygen level was gradually decreased from 21% to 8% over one week and animals were kept at 8% oxygen for another 7 days. After two weeks animals were euthanized using CO_2_ and total RNA was isolated from each quadriceps femoris (QF) muscle by Tri-Reagent (Ambion, Austin, TX). Microarray analysis was then performed using an Affymetrix Mouse Genome 430 2.0 Array according to the standard Affymetrix protocol (Affymetrix, Santa Clara, CA). Data were then quantile normalized using the R library gcrma [19]. Differentially expressed genes between normoxic and hypoxic mice were identified using SAM analysis [32]. Genes that have an FDR smaller than 10% were used to map to the neighborhood of the VO2max in the COPD network. Genes linked to the GO term “ chromatin remodeling” (represented in Figure S8 in Text S1) were identified using the web-based tool DAVID [26].

## Results

### Integration of physiological and molecular responses acquired in the 8 weeks training study within a network inference framework

With the aim of developing an initial high level model representing the relationships between systemic and local signals we set to integrate all measurements available (physiology, blood cytokine levels and muscle gene expression measurements) for the individuals recruited in the 8 weeks training study. These samples included both pre and post-training blood and biopsy samples.

We then tested the individual network modules generated using the hubs in Table S3 in Text S1 for enrichment in GO and KEGG functional terms, which revealed a remarkably coherent association between target genes and the biological functions of network hubs. Neighbourhoods of physiological variables, such as VO_2_ peak, were mainly enriched in glycolysis/gluconeogenesis, mitochondria respiratory chain, oxidative phosphorylation (all positively correlated to VO_2_ peak), and components of the ribonucleoprotein complex and RNA processing (negatively correlated to VO_2_ peak, Figure S2 in Text S1 and Table S4 in Text S1). Genes associated with mRNA expression of cytokine and growth factor receptor hubs were enriched in functions related to tissue remodelling and immune response (generally positively correlated with the expression of receptors for growth factors and cytokines, Table S5 in Text S1), while genes associated with expression of glycolysis/gluconeogenesis enzymes were mainly enriched in energy metabolism-related functions (Table S6 in Text S1). Systemic inflammatory cytokines were weakly connected to the network (only three significant correlations with genes linked to physiological measurements).

**Figure 1:**
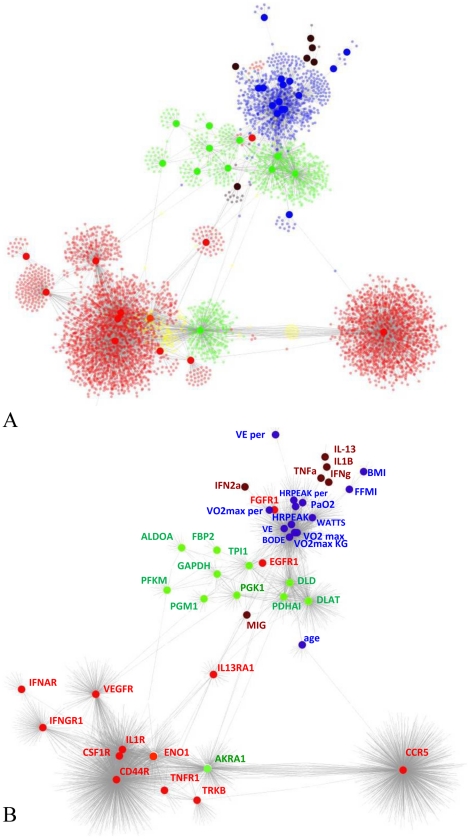
Network integrating multi-level measurements in the 8 weeks training study. The figure shows the network representing the relationship between physiological measurements, serum cytokine levels and mRNA expression profiles in the muscle, following ARACNE analysis of the 8 weeks training dataset. Panel A shows an outline of the network topology where individual network hubs have been annotated. Red nodes represent genes coding for receptors for growth factors and immune system mediators, blue nodes represent physiological measurements, brown nodes represent serum cytokines, and green nodes represent gene expression of enzymes in the glycolysis and gluconeogenesis pathways. Panel B shows all nodes in the network (representing 16350 genes or measurements). The colour of nodes follows the same code as panel A. In addition yellow nodes represent genes in common between cytokine and growth factor and glycolysis/gluconeogenesis network hubs.

The union of the individual modules and consequent visualization with a force driven layout revealed a clear separation between two main sub-networks. First, a large sub-network, primarily representing the association of modules built from cytokine (both plasma and skeletal muscle) and growth factor receptor hubs and enriched by tissue remodelling functions. Second, a sub-network representing the association of modules built with physiology and skeletal muscle glycolysis/gluconeogenesis hubs and enriched in energy metabolism genes (Figure 1). Within this sub-network we found that many of the physiological variables (PaO2, HRPEAK, HRPEAKper, WATTS, VE, VO2max, VO2maxper, VO2maxkg, BODE) were highly correlated (Figure S3 in Text S1) and consequently clustered together (Figure S4 in Text S1) to form a very compact network module. The other physiological variables (VEper, FFMI, BMI and age) were separated from this module and were characterized by a limited number of connections with gene expression variables (30 genes) (Figure S4 in Text S1).

### Uncoupling between expression of tissue remodelling and bioenergetics modules is a specific feature of skeletal muscles of COPD patients

**Figure 2:**
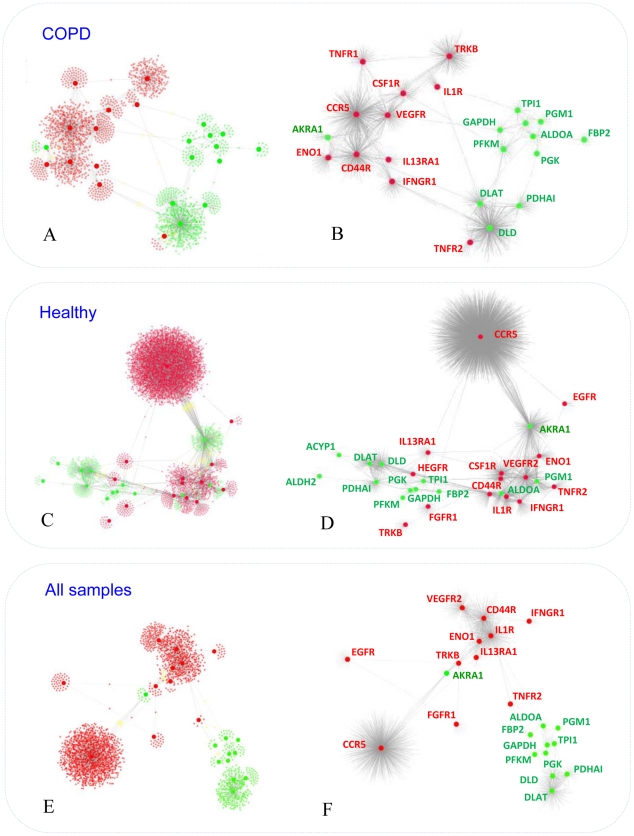
Healthy and COPD specific networks. The figure shows interaction networks built using solely expression profiling data representing healthy (panels A and B) or COPD (panels C and D) muscle biopsy mRNA profiles. For comparison with Figure 1, the network representing integrated healthy and COPD samples has been represented in Panels E and F. Figure layout and the colour coding are identical to Figure 1.

In order to assess whether the separation between tissue remodelling and bioenergetics sub-networks observed in the network constructed by integrating all measurements in the training study may be a specific feature of COPD, it was necessary to developed two separate networks representing healthy and diseased muscles (Figure 2).

Our analysis, further supported by the integration of an additional dataset representing an independent and comparable clinical study (see Methods section for details), confirmed that the separation between cytokine/growth factor receptors and bioenergetics modules is a specific feature of COPD networks (Figure 2A, 2B) (Figure S5 in Text S1). In contrast, there was extensive overlap between the neighbourhood of receptors and intermediate metabolism in the network constructed from healthy muscle biopsies (Figure S2C, S2D and Figure S3 in Text S1).

In order to assess whether uncoupling was a general feature of pathological muscles, network models representing the transcriptional state of dystrophy (Figure S6A and S6B in Text S1) and diabetic muscles (Figure S6C and S6D in Text S1) were also developed. In both cases the cytokine/growth factor receptor and bioenergetics sub-networks were localized in close proximity. It was remarkable that even in a situation where muscle functionality is severely impaired by the lack of important structural components, a condition typical of muscle dystrophy, energy and tissue remodelling functions still remain co-ordinately regulated. This observation further strengthens the concept that uncoupling between muscle remodelling pathways and bioenergetics is a specific feature of COPD muscles.

### The molecular response to endurance training highlights a failure of diseased muscles to transcriptionally regulate tissue remodelling and bioenergetics

Healthy individuals responded to training by modulating expression of a relatively large number of genes (3908, Figure S7A in Text S1) compared to COPD_N_ (953, Figure S7B in Text S1) and to COPD_L_ patients (6 genes). There was a significant overlap (36%, FDR<10%) at the gene level and an almost complete overlap at the pathway level (Figure S7 in Text S1) between the response to training in COPD_N_ and healthy subjects whereas none of the six genes differentially regulated in the COPD_L_ group were modulated in healthy and COPD_N_ individuals (Figure S7C in Text S1).

**Figure 3:**
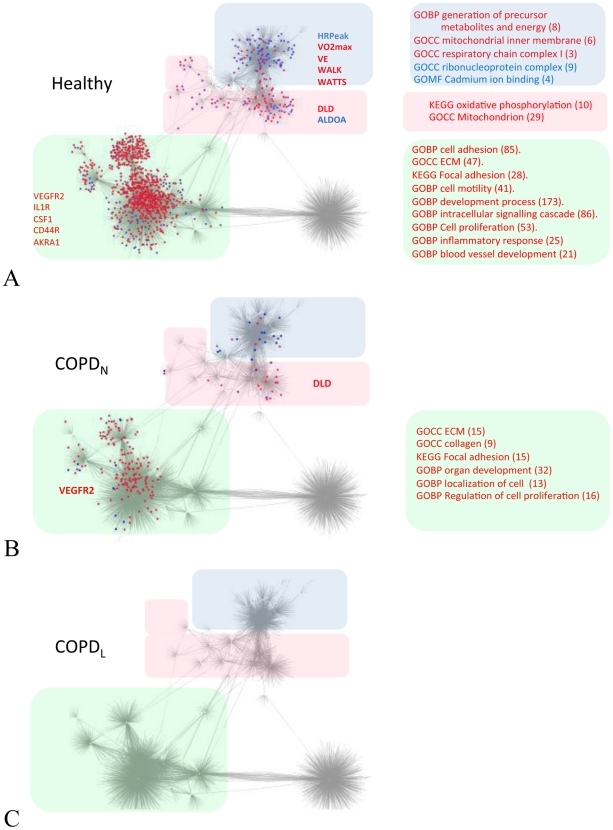
Training response of healthy and COPD individuals visualized on the integrated 8 weeks training network. The networks represent the genes up-regulated (marked in red) and down-regulated (marked in blue) mapped on to the interaction map inferred using the 8 weeks training multi-measurement dataset (shown in detail in **Figure 1**). Panel A shows genes differentially regulated after training in healthy individuals. Panel B shows genes differentially expressed after training in N-BMI COPD patients. Network hubs representing genes up-regulated (red) or down-regulated (blue) are listed on the side of the relevant sub-networks. The drastic reduction in the molecular response to training associated with the cytokine and bioenergetics clusters is evident. Panel C shows the lack of response to training in COPD_L_ patients.

In order to identify which part of the network was modulated in response to training, these differentially expressed genes were mapped onto the integrated network described previously (Figure 1). Genes that were up-regulated after training appear to cluster primarily in proximity to receptors for the cytokines IL-1 (IL1R), CSF1 (CSF1R), plasminogen (ENO1) and the key receptor for the pro-angiogenesis growth factor VEGF (VEGFR2) (Figure 3A). A second cluster that is also predominantly populated by genes up-regulated in response to training represents the neighbourhoods of glycolytic enzymes (Figure 3A) and to the epidermal growth factor receptor (EGFR). In contrast, genes that are either up or down-regulated in response to training populated the neighbourhood of physiological variables, representing energy (up regulated) and mRNA processing (down-regulated) functions. The transcriptional response of COPD_N_ muscles to training followed a very similar pattern although as reported in Figure S5C in Text S1, the number of genes involved was much lower (Figure 3B). As mentioned previously, COPD_L_ patients do not respond to training with a detectable change in the transcriptional state of the muscle (Figure 3C). In addition to the transcriptional uncoupling described above, the transition between healthy and diseased individuals is therefore characterised by a reduction in the ability to transcriptionally regulate expression of genes in both tissue remodelling and bioenergetics pathways.

**Figure 4:**
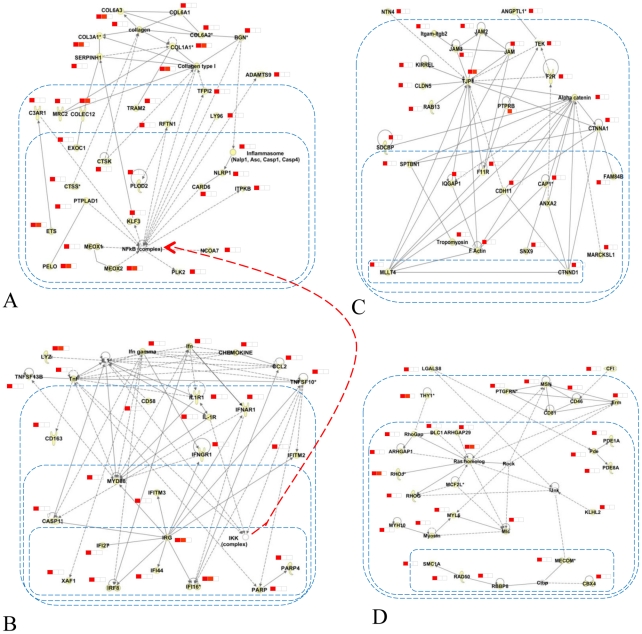
Ingenuity networks regulated by endurance training. This figure shows the most important findings derived from Ingenuity analysis using genes that are differentially expressed in response to training in healthy individuals and connected to receptor hubs in the previously inferred network. Panel A and Panel B represent two interconnected networks linked the transcription of several cytokines (IL1, TNFα, IFNγ, CCL2) and activation of the NF-kB complex (Panel A), leading to the activation of several NF-kB targets related to connective tissue formation (Panel B). Panel C shows the relationship between upregulation of gap junction complexes (JAM, JAM2, JAM3 and TJP1) and activation of important structural components of muscle fibres (e.g. tropomyosin). Panel D shows the important functional link between activation of several Rho GTPases and muscle development, represented by genes encoding for a component of the hexameric ATPase cellular motor protein myosin (MYL5) and MYH10.

To better characterize molecular networks associated with the growth factor and cytokines components of the network (see Figure 1 and Figure 2) directly connected to receptor hubs and differentially expressed in response to training in healthy individuals (predominantly located in the lower part of the network), we selected and used these genes as input for the Ingenuity Pathway Analysis (IPA) software. The analysis identified 17 networks (see method section for details of the procedure) (Table S7 in Text S1). Among the most significant findings, two interconnected networks linked the transcription of several cytokines (IL1, TNFα, IFNγ, CCL2) and activation of the NF-kB complex (Figure 4A), leading to the activation of several NF-kB targets related to connective tissue formation (Figure 4B). Figure 4C shows the relationship between upregulation of gap junction complexes (JAM, JAM2, JAM3 and TJP1) and activation of important structural components of muscle fibres (e.g. tropomyosin). The network in Figure 4D shows the important functional link between activation of several Rho GTPases and muscle development, represented by genes encoding for a component of the hexameric ATPase cellular motor protein myosin (MYL5) and (MYH10).

Although not all captured by our network model, 6 myogenic markers were found to be regulated in response to training (Table S8 in Text S1). Five of these were up-regulated. Consistent with what was previously observed (above), the training-induced expression of all the genes represented in these networks was defective in COPD individuals (Figure S4 and Table S8 in Text S1).

### Interleukin-1 promotes activation of tissue remodelling *in vivo* and mirrors the effect of training in healthy human subjects

Because of the strong correlation between expression of IL-1 receptor and genes involved in muscle remodelling and the networks identified by the Ingenuity analysis, we reasoned IL-1 might be responsible for inducing a significant component of the transcriptional response to training observed in healthy individuals. In order to test this hypothesis we performed intra-venous injections of IL-1β in mice, and characterised the transcriptional response in both glycolytic and oxidative muscles using microarray expression profiling.

The response of mice to injection of IL1β supported our initial hypothesis. IL-1β treatment induced within 24 hours the up-regulation of 336 genes in oxidative muscle, whereas in glycolytic muscles it induced the up-regulation of 263 genes and the down-regulation of 201 genes (Table 1). Genes up-regulated in both muscle fibre types were enriched for structural components of the sarcomere (ACTA1, NEB, CRYAB, ANKRD2). Apart from this, the response of the two muscle fibre types was very different. Oxidative muscles up-regulated genes encoding for components of the extracellular matrix and organ development including angiogenesis (SOX18, VEGFA, SERPINF1, ANXA2, PTEN, ENG), whereas glycolytic muscles modulated genes involved in oxidative phosphorylation (COX8A, COX5A, NDUFB8, NDUFB5, NDUFS4, NDUFV2) and ribosomal components (Ribosomal protein chains 10A,s5 and I37A, and mitochondrion ribosomal protein chains 35 and 45).

**Table 1:** Response to IL1 injection in oxidative and glycolityc muscle.

Tissue type	sign	#	Functional annotation
Oxidative muscle	up	257	**GOCC ECM (64). GOCC sarcomere (7).** GOCC lysosome (8). GOBP anatomical dev structure (41). GOBP angiogenesis (6). GOMF oxidoreductase activity (18).
Glycolitic muscle	up	170	**GOCC myosin complex (5). GOCC sarcomere (6).** GOBP generation of precursor metabolites and energy (13). KEGG oxidative phos. (8) GOCC mit (20). GOBP gluconeogenesis (5).
Glycolitic muscle	down	150	GOCC extracellular space (23). GOBP protein transport (10). GOBP metabolic process (60). GOCC Mit (4)

Functional annotation of genes changing in response to IL1 injection in oxidative and glycolityc muscle. These functions are not necessarily significantly enriched.

The high percentage of differentially expressed genes encoding for tissue remodelling functions (extracellular matrix components, organ structure development, cell communication) and bioenergetics (oxidative phorphorylation) which included the oxygen sensitive regulator HIF-1, suggested that IL-1β has the potential to reproduce a component of the physiological transcriptional response to training that is defective in COPD patients.

**Figure 5:**
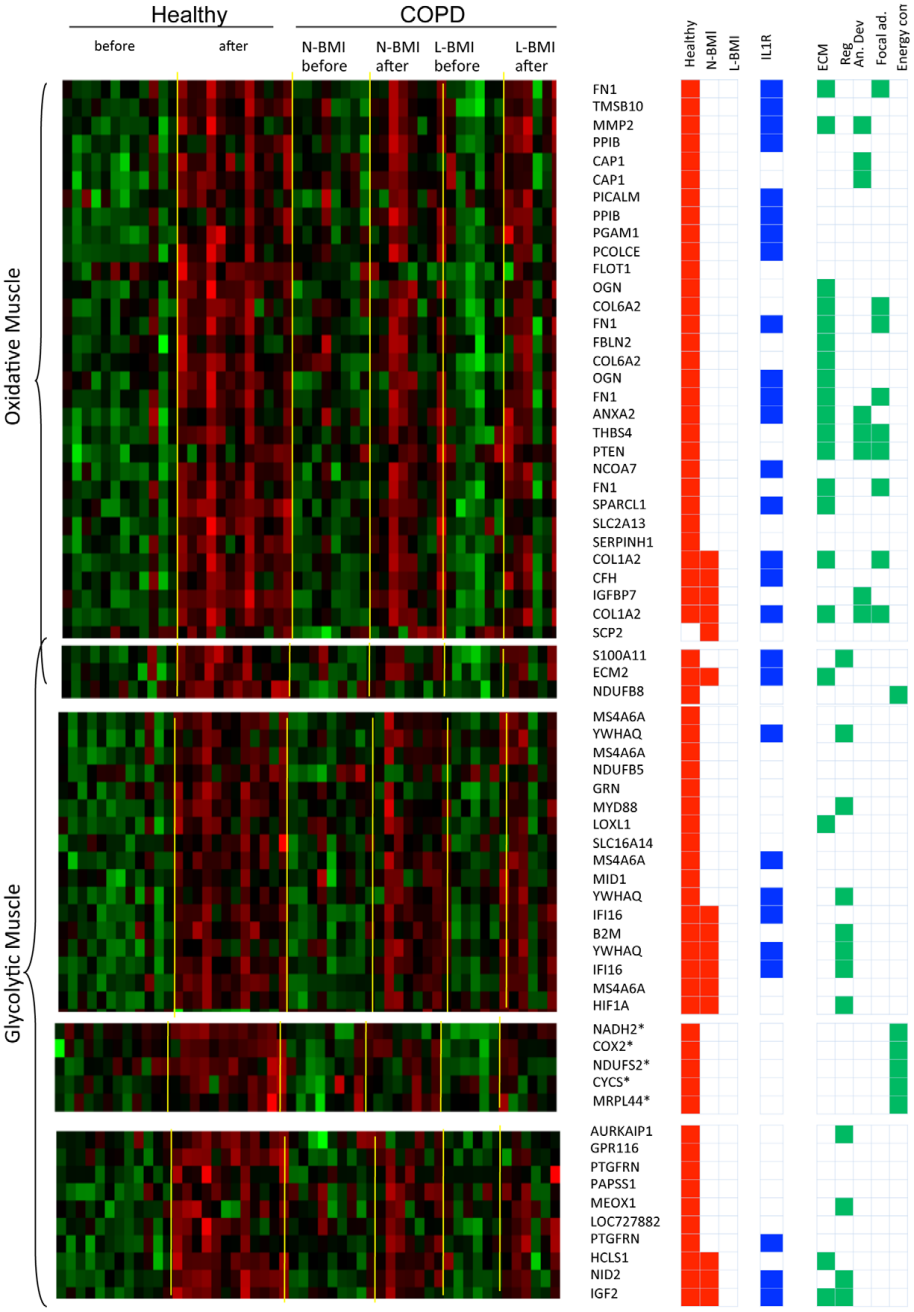
Effects of IL-1β in mouse glycolytic and oxidative muscles. This figure describes the effects of recombinant interleukin 1 on the transcriptional state of glycolytic and oxidative muscles. Panel A represents the heatmap of expression profiles in human muscle biopsies of genes that are differentially regulated in response to training in humans and also induced by IL1β in mouse muscles. Panel B shows the localisation of genes up-regulated in oxidative and glycolytic muscles on the COPD interaction network represented in Figure 1.

In order to assess this hypothesis, we mapped genes differentially expressed in the IL-1 β mouse experiment on the human interaction network represented in Figure 1. 78% and 68% (265 and 317 genes) of the genes modulated in response to IL-1β treatment in oxidative muscle and glycolytic muscle, respectively, in mice mapped on to the human dataset. Of these, 34 genes were up-regulated after endurance training in healthy individuals (Figure 5). A significant proportion of these genes were, in oxidative muscle, also directly connected with the receptor for Interleukin 1 in the integrated network model. This observation further validates the model and supports a role for IL-1β in the physiological response to training.

### Decreased training-induced expression of NF-kB target genes in COPD muscles

The lack of activation of tissue remodelling functions in COPD muscles, including the component linked to IL-1β signalling, may be a consequence of the inactivation of myogenic pathways due to over-activation of NF-kB signalling induced by chronic exposure of pro-inflammatory cytokines [14]. We reasoned that if this hypothesis were correct, we should observe over expression of a number of NF-kB target genes in diseased muscles.

We had previously observed that a number of NF-kB targets, identified by the IPA software in the ‘tissue remodelling’ section of the network, were up-regulated during training in healthy individuals, but failed to respond in COPD patients (Figure 4A). Although interesting, this observation was based on a loose definition of NF-kB targets, as many connections reported in the Ingenuity database are indirect. We therefore further tested the NF-kB over-activation hypothesis by analyzing expression of 94 experimentally validated targets of NF-kB (Table S9 in Text S1). Against the working hypothesis, we could not identify any NF-kB target gene differentially regulated between normal and diseased muscles. On the contrary, we could detect 13 genes that were differentially regulated in response to training in healthy individuals (all up regulated, 10%FDR and fold>1.5) but not in COPD individuals with normal or low BMI (Table 2). These observations clearly do not support the over-activation hypothesis. On the contrary, they suggest that in COPD muscles training-induced activation of NF-kB is repressed.

**Table 2:** NFKB Targets in response to training in healthy muscle.

genes	FOLD CS-CT	FDR
TFPI2	1.88	0.02
PLAU	1.62	0.02
CD74	1.60	0.03
CCL2	1.69	0.04
ENG	1.41	0.04
CCND1	1.45	0.07
NQO1	1.69	0.08
CD44	1.80	0.09

NFKB Targets which are significantly different in response to training in healthy patients.

### Expression of epigenetic histone modifiers discriminates between healthy and diseased muscles and is correlated with peak oxygen consumption

In order to explore an alternative hypothesis that may explain muscle wasting in COPD patients, we performed an unbiased analysis to identify functional pathways differentially modulated between healthy and diseased muscles. We therefore used two-factor ANOVA, taking into consideration both disease and training.

**Figure 6:**
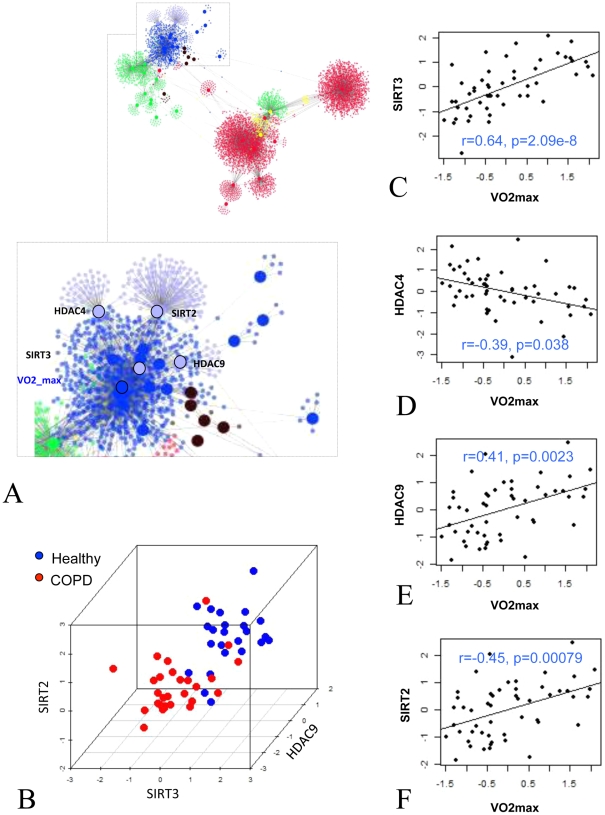
Expression of chromatin modification enzymes in COPD and healthy muscles. Panel A represent the close localization of histone deacetylase enzymes to VO_2_max in the inferred network. Panel B is a 3D plot representing the expression of histone deacetylase enzymes and the separation between disease and healthy muscles. Panel C, D, E and F represent the scatterplots between VO_2_max and the expression of histone deacetylase enzymes.

Consistent with the analysis described above, we identified tissue remodelling and energy-associated pathways as significantly differentially expressed for both factors analysed (Table S10 in Text S1). However, one additional category of 20 chromatin modification enzymes were also differentially expressed (Table 3). Among these we could identify four histone deacetylase enzymes, which are known to be particularly relevant for controlling expression of muscle differentiation (HDAC9 and HDAC4, SIRT2) and bioenergetics (SIRT3) related genes. Consistent with their potential role in COPD muscle wasting, expression of these genes was sufficient to discriminate between diseased and healthy muscles with great accuracy (90% of cross-validated prediction accuracy using a K-nearest neighbour - model) (Figure 6A). By mapping them on the network model described in Figure 1 we discovered that they localized close to VO_2_peak, (Figure 6B) suggesting that their transcription may be up-regulated by oxygen availability and/or oxidative capacity in the skeletal muscle.

**Table 3:** Functional annotation of genes identified by two-factor ANOVA.

Functional Category	Genes in the Function ( 10% FDR)
GOCC Chromatin remodelling complex (disease factor)	APPL1, ARID1A, ARID1B, CHD4, CIR1, HDAC4, HDAC7, HDAC9, MBD2, MTA1, PHF21A, RSF1, SIN3A, SIRT2, SIRT3, SMARCA4, SMARCB1, SNARCE1, SUDS3, TBL1XR1
GOCC histone deacetylase complex (exercise factor)	APPL2, CHD3, CHD4, GATAD2A, HDAC1, HDAC11, HDAC3, HDAC4, HDAC5, LOC642954, MBD3, NRIP1, RBBP4, RBBP7, RERE, SAP18, TAL1, TBL1XR1

Functional enrichment of genes that are significantly differentially expressed in both disease and training.

### Genes predicted to be linked to VO2max ARACNE are induced in a mouse model of chronic hypoxia

In order to elucidate whether genes linked to VO2max in our network model are part of the physiological response to hypoxic conditions we analyzed a public domain microarray study developed by Budak *et al.* (GEO: GSE9400) [31] which represents the response of murine skeletal muscles to 2 weeks hypoxic conditions.

**Figure 7:**
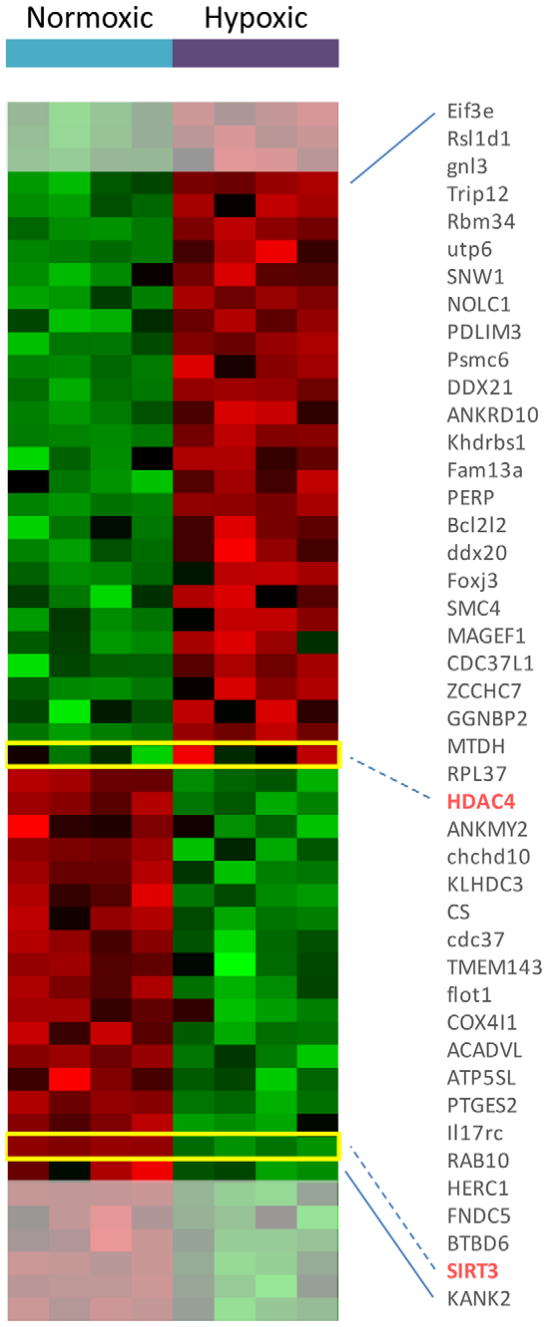
Genes represented in the neighborhood of VO2max are transcriptionally regulated in a mouse model of hypoxia. This heatmap represent the expression of genes in the neighborhood of VO2max that are also differentially expressed in skeletal muscles, in a mouse model of hypoxia. It shows that the majority of these genes (82%) are regulated in the direction predicted by the network we developed (Figure 1). The HDAC4 and SIRT3 genes are among these and are highlighted in red. Genes that are not modulated in the direction we predicted are shaded in grey.

We discovered that 45% of genes connected to VO2max in the network model were transcriptionally regulated in the mouse model of hypoxia and that a striking 82% of these were regulated in the direction predicted by the network analysis (Figure 7). Among these, we found the genes encoding for the chromatin modifiers HDAC4 and SIRT3. HDAC4 is up-regulated, whereas SIRT3 is down-regulated in hypoxic mice compare to normal (Figure 7), which is consistent with the sign of the correlation with VO2max observed in the clinical study (Figure 6A and 6C). Interestingly, we also discovered that many other genes encoding for chromatin modifiers were differentially expressed in response to hypoxia (Figure S8 in Text S1).

## Discussion

The networks we have developed represent the first model linking molecular and physiology measurements in skeletal muscle of COPD patients. It provides convincing evidence that a failure to co-ordinately activate expression of several tissue remodelling and bioenergetic pathways is a specific landmark of diseased muscles. Moreover, our model is consistent with the view that the abnormal expression of a number of histone modifiers potentially regulated by oxygen availability may be responsible for alterations in both tissue remodelling and bioenergetic functions. This hypothesis has important implications as it places cell hypoxia and oxidative capacity as the main drivers for skeletal muscle abnormalities in COPD patients.

### Response to training in healthy and diseased muscles

We identified several pathways which are transcriptionally regulated in healthy individuals in response to training. These involve the up-regulation of several tissue remodelling genes/pathways (Plasminogen receptor, VEGF, pro-inflammatory signals such as IL1) as well as modulation of energy and ribosome biogenesis functions. Without exception, the exercise-induced modulation of these pathways is severely impaired in COPD individuals.

The Ingenuity pathway analysis has revealed several detailed mechanisms associated with the inflammation and growth factor receptor component of the network we have inferred. Two of these networks (Figure 4A and 4C) are part of a larger network linking the effect of pro-inflammatory signals such as IL-1, TNFα and IFNγ to the training induced up-regulation of several components of the extracellular matrix in healthy subjects. Although circulating concentration of IL-1β are largely unaffected by exercise there is evidence of increased local IL-1β levels within skeletal muscle, likely in response to micro-injury of skeletal muscle with increased activity [27]. The *in vivo* experiment we have performed show that indeed a component of the transcriptional response observed in healthy individuals and defective in COPD patients may be mediated by interleukin-1β. Moreover, we found that mouse glycolytic and oxidative muscles respond similarly to IL-1β in respect to the up-regulation of structural components of the muscle but diverge in the regulation of genes involved in energy metabolism and ubiquitination (up-regulated in glycolytic muscle), or extracellular matrix and tissue remodelling including angiogenesis (up-regulated in oxidative muscles).

Patients with mild to moderate COPD have a greater proportion of fatigue-susceptible anaerobic (glycolytic Type II) relative to fatigue-resistant aerobic (oxidative Type I) fibres, suggesting a slow-to-fast transition. It has been proposed that <27% Type I and >29% Type IIx fibres offers a pathological threshold for COPD [33]. This is consistent with a shift towards a more glycolytic enzyme profile, and would contribute to an increase in skeletal muscle fatigability [34]. Although individual fibre phenotypes are well conserved among mammals virtually all human skeletal muscles are of mixed composition, hence mouse muscles with discrete metabolic profiles were used to identify differential responses to IL-1β according to metabolic type.

A mechanistic link between the activity of these tissue remodeling pathways and myogenesis is well supported by the current literature. For instance, the plasminogen receptor ENO1 has been demonstrated to be an important component of skeletal myogenesis by concentrating and enhancing plasmin generation of the cell surface [35]. Interestingly, ENO1 KO mice show severe defects in muscle regeneration following injury [31]. Components of the extracellular matrix, which are induced by signaling from many of the receptors present in the network are also mechanistically linked to myopathies. For example, Col6a1–deficient and Col15a1-deficient mice have a muscle phenotype that strongly resembles human myopathies [36], [37].

Inflammatory and chemo-attractant mediators are also known to be key factors in driving muscle remodelling in the normal physiological response to training [38] and in response to trauma [35]. In the latter, IL1β also promote phagocytosis of trauma-induced cellular debris by macrophages, which themselves can continue to secrete this cytokine up to 5 days post-injury [39]. CCR2 null mice with cardiotoxin induced injury has been shown to have a delayed angiogenesis and VEFG production compared to wild type mice with muscle fibre size increase observed only after restoration of tissue VEGF [40]. Other studies have also shown the crucial role of VEGF in angiogenesis and in muscle regeneration [41], [42].

There is also strong evidence that bioenergetics and tissue remodelling pathways are linked [43]. It has been shown that genes coding for components of collagen V and collagen VI play an important regulatory role in ECM maturation, where reduced expression promotes apoptosis, mitochondrial dysfunction and muscle degeneration [43]. Other studies have shown that bioenergetics knockouts, such as H6PD null mice is responsible for inducing severe skeletal myopathy by altering sarcoplasmic reticulum redox state [44].

### Decreased training induced expression of NF-kB target genes in COPD muscles

A number of *in vitro* and *in vivo* studies have been used in the past to support the hypothesis that a systemic inflammation-driven mechanism leads to inactivation of myogenic pathways in COPD muscles. Our analysis is the first attempt to challenge this hypothesis using genome-wide data, and in a clinically relevant setting. We could not find evidence of over-expression of NF-kB target genes in COPD muscles. On the contrary, we saw that a subset of direct (Table 2) and indirect targets (Figure 4A) of NF-kB were up-regulated in healthy individuals in response to training but not in COPD patients (*p*<0.05). These results are consistent with a recent observation demonstrating failure to activate NF-kB in response to acute training in a small subset of COPD patients [15]. Taken together, these results suggest that training-associated inactivation rather than over activation of NF-kB may be a feature of diseased muscles.

Additional observations also argue against a primary role of chronic inflammation in muscle wasting. For example, although we clearly have identified increased levels of cytokines in COPD patients (Figure S1B in Text S1) with respect to normal individuals, we could find little correlation between the concentration of these cytokines and the muscle transcriptional state, implying that these signals may have a smaller effect on muscle physiology than previously thought. This latest observation is consistent with previous reports showing that TNFα levels measured in COPD muscles were not significantly higher than in healthy muscles [45].

### Is there an epigenetic basis for muscle wasting in COPD?

Since the NF-kB over-activation hypothesis is unlikely to explain the inhibition of muscle remodelling observed in COPD, can we propose an alternative mechanism for muscle wasting in COPD?

There are several pieces of evidence in favour of the hypothesis that an imbalance in expression of oxygen-correlated chromatin modifying enzymes, which we have shown to be a landmark of COPD muscles, could explain failure to modulate both tissue remodelling and bioenergetic functions in response to training. In Figure 6 we have shown that the expression of SIRT2, SIRT3, HDAC4 and HDAC9 is sufficient to discriminate healthy and diseased muscles. At the individual gene level, healthy muscles are characterized by a higher expression of HDAC9, SIRT3 and by a lower expression of HDAC4 (Table 4) with respect to COPD muscles.

**Table 4:** Chromatin remodeling complex genes in healthy and disease muscle.

Gene Symbol	Fold Disease vs. Healthy	ANOVA Factor Disease (FDR)
HDAC9	2.30	6.61e-5
HDAC4	−1.48	0.05
SIRT2	1.27	0.0016
SIRT3	1.31	0.02

Genes that are significantly different between healthy and disease belonging to the functional category of GOCC Chromatin remodeling complex.

The role of HDAC9 and HDAC4 in muscle development is well documented [46]. For example, HDAC9 is a transcriptional repressor involved in feedback control of muscle differentiation, acting in concert with MEF2 to repress activity-induced genes [47], while HDAC4 is up-regulated in pathological conditions such as muscle denervation [48], and it has been described to be a critical regulator of muscle atrophy by activation of E3 ubiquitin ligases [49]. It is possible, therefore, that a lower expression of HDAC9 and a higher expression of HDAC4 in COPD muscles may be linked to a reduced ability to activate muscle remodelling. Abnormal expression of SIRT2 may also contribute to a failure to activate an appropriate muscle remodelling response. SIRT2 is a NAD^+^-dependent histone deacetylase that regulates muscle gene expression and differentiation by forming a complex with MyoD [50]. When over-expressed, this retards muscle differentiation. Conversely, cells with decreased SIRT2 differentiate prematurely. Interestingly, the activity of SIRT2 is dependent on the redox state of the cell [50], which showed evidence of being abnormal in COPD muscles [11], [12], [13]. In the neighborhood of SIRT2, we have also identified TXN2 that is known to play an important role in protection against oxidative stress [51]. Similarly, GAB1 is shown to play a role in oxidative stress signaling [52] and it is identified in the neighborhood of HDAC9 in the network. Since changes in ROS production are known to influence the expression of HDACs [53], the network we have identified may represent this important control mechanism.

SIRT3 is a NAD^+^-dependent histone deacetylase that may account for the characteristic loss of transcriptional modulation of bioenergetic genes in response to training in COPD muscles. SIRT3 is localized in the mitochondrial matrix, where it regulates the acetylation levels of metabolic enzymes, including acetyl coenzyme A synthetase 2 [54], [55]. Mice lacking both SIRT3 alleles show hyperacetylation of several mitochondrial proteins, associated with decreased levels of fatty-acid oxidation, and display a selective inhibition of electron transport chain Complex I activity leading to reduction in basal levels of ATP in several organs [56]. These and other data implicate protein acetylation as an important regulator of mitochondrial function *in vivo*, and it is therefore feasible that an altered expression of SIRT3 in the muscles of COPD individuals may contribute to the observed imbalance in mitochondria functionality. It is interesting that SIRT3 in our model is positively correlated with VO_2_peak, suggesting that the lower levels of expression of this enzyme observed in COPD muscles may be the consequence of tissue hypoxia. Consistent with this, histone deacetylase (HDAC) inhibitors reduce HIF-1α protein expression leading to down-regulation of VEGF and other angiogenesis-related genes [57], potentially explaining the reciprocal relationship between extent of muscle capillarity and the degree of COPD [58]. The abnormal expression of a relatively small number of histone modifying enzymes could therefore account for a wide spectrum of abnormal responses observed in the muscles of COPD patients, and may also explain the limited efficacy of training as a therapeutic option.

This view is supported by our observation that indeed hypoxia induces modulation of a number of chromatin modifiers in a mouse model of chronic hypoxia (Figure S8 in Text S1) and that indeed SIRT3 and HDAC4 are among them (Figure 7).

### Conclusion

Our work represents the most accurate system level representation of COPD muscles to date. Further work is, however, needed to elucidate the precise mechanism for muscle inactivation. If the mechanism for muscle wasting suggested by our observations on HDACs were to be validated in a clinical setting this would open up a very exciting therapeutic avenue. The use of non-toxic histone deacetylase inhibitors such as valproate has already shown promising in treatment of haematological cancer [59], [60], [61], and may help to restore mitochondrial functionality and the ability to activate muscle remodelling in COPD patients. In this context it is possible that the appropriate pharmacological regime coupled with physical rehabilitation may lead to recovered muscle functionality, and improved quality of life.

## Supporting Information

Text S1 **Supplementary material.** This file contains supplementary figures (Figures S1–S8) and tables (Tables S1–S10).
(DOC)
