# Supplementary material for: A Systems Biology Approach Identifies Molecular Networks Defining Skeletal Muscle Abnormalities in Chronic Obstructive Pulmonary Disease
Source: PLoS Comput Biol. 2011 Sep 1;7(9):e1002129. doi: 10.1371/journal.pcbi.1002129 (PMC3164707; doi:10.1371/journal.pcbi.1002129)
Supplement: Text S1 — Supplementary material. This file contains supplementary figures (Figures S1–S8) and tables (Tables S1–S10). (DOC) [file pcbi.1002129.s001.doc]

**Figure S1. 8-weeks training study: Disease associated cytokines and Physiological measurements.**

The figure shows two heatmaps representing respectively the standardized values for the physiology and serum cytokine measurements. Data are standardized to represent above average values in red and below average values in green. Panel A represents significantly different physiological measurements in the disease groups (Healthy, L-BMI and L-BMI). Those variables that are also significantly changing in response to training are labeled in red. Panel B represent serum cytokine measurements significantly different in the disease groups (Healthy, N-BMI and L-BMI). No cytokines were significantly different in response to training.

**Figure S2. Correlation between VO2max and its neighbourhood as inferred by ARACNE.**

Panel A represents the functional annotation of the genes, which are positively and negatively correlated with VO2max. Panel B shows instances of genes belonging to mRNA export from nucleus, mRNA processing, and Ribosome functional terms. Panel C shows instances of genes that belong to Glycolysis/Glycogenesis and to mitochondrion respiratory chain functional terms.

**Figure S3. Correlation between physiological variables.**

This figure shows the scatterplots between physiological variables that are highly co-regulated and form a compact subnetwork.

**Figure S4.** **Detailed representation of the physiological subnetwork in the COPD network.**

This figure is a detailed representation of Figure 1 in the manuscript , representing the subnetwork of physiological variables.

**Figure S5. Healthy and COPD specific networks.**

The figure shows the networks obtained by ARACNE analysis with healthy (panel A) and COPD (panel C) specific datasets (see methods section for details). Panel B and Panel D show the correlation between two landmark genes (IL1R and ALDOA) in the 8 weeks training study for healthy and disease sample biopsies respectively. Note that the network models represent the lack of correlation between tissue remodelling and bioenergetics in COPD patients.

**Figure S6. Networks representing diabetes and dystrophy muscles.**

Panel A and Panel B represent the network inferred from the dystrophy dataset (see methods for details). Panel C and D represent the full interaction networks inferred from the diabetes dataset (see methods section for details). Nodes marked in red represent the neighborhood of receptors of the immune receptors; green nodes represent the neighborhood of central metabolism hubs. From a simple visual inspection it is evident in the two networks that the intermediate metabolism genes are closely linked to the expression of inflammation and growth factor signals.

**Figure S7. 8-weeks training study: Expression profiling analysis of muscle biopsies.**

The figure summarizes the results of the microarray analysis of muscle biopsies representing response to endurance training. Panel A shows the heatmap representing genes differentially regulated in response to training in healthy individuals. Panel B shows the heatmap representing genes differentially regulated in response to training in N-BMI individuals. Functions enriched in the up and down regulated genes are labelled on the right side of the heatmap and colour coded to discriminate between up-regulated (red) and down-regulated (green) functions. Panel C display a Venn diagram showing the overlap between differentially regulated genes in response to training in healthy, N-BMI and L-BMI subjects. It is evident that N-BMI and L-BMI show a progressively less pronounced response to training.

**Figure S8. Differentially expressed genes in the hypoxic mouse model.**

This figure represents the genes belonging to chromatin modifier related gene ontology terms that are significantly different in hypoxic mouse model.

**Table S1. Physiology measurements showing the effect of endurance training in healthy and COPD individuals**

|  | | **COPD patients** | **Healthy subjects** |
| --- | --- | --- | --- |
|  | |  |  |
| **VO_2_ peak, ml/min^-1^** | **Pre** | 896±250 | 1566±410 |
|  | **Post** | 1076±381 | 1819±533 |
|  | **Δ post-pre** | 180±183† | 253±204† |
| **VO_2_ peak, %pred** | **Pre** | 69±20 | 88±15 |
|  | **Post** | 84±25 | 102±19 |
|  | **Δ post-pre** | 15±11‡ | 14±11† |
| **Wpeak, watts** | **Pre** | 56±21 | 111±27 |
|  | **Post** | 73±29 | 135±37 |
|  | **Δ post-pre** | 17±11‡ | 24±13‡ |
| **6MWT, meters** | **Pre** | 443±123 | 585±87 |
|  | **Post** | 472±132 | 633±68 |
|  | **Δ post-pre** | 29±25‡ | 48±51* |

**Table S2. Serum and cytokine growth factors measured using Luminex technology.**

| IL-1beta | IL-6 | IL-15 | MIP 1alpha | MCP-1 |
| --- | --- | --- | --- | --- |
| IL1RA | IL-7 | IL-17 | MIP 1beta | VEGF |
| IL-2 | IL-8 | TNF alpha | IP-10 | G-CSF |
| IL-2R | IL-10 | IFN alpha | MIG | EGF |
| IL-4 | IL-12 | IFN gamma | Eotaxin | FGF-basic |
| IL-5 | IL-13 | GM-CSF | RANTES | HGF |

**Table S3: List of physiology, serum cytokines and immune system/growth factors receptors gene expression measurements used as network hubs in the ARACNE procedure.**

**Table S4. COPD interaction network**

Representative functional terms enriched in the neighbourhood of Physiological variables

**Table S5. Representative functional terms enriched in the neighbourhood of immune system and growth factor receptor hubs in the COPD interaction network.**

**Table S6. Representative functional terms enriched in the neighbourhood of central metabolism hubs in the COPD interaction network.**

Table S7. Top networks identified by the Ingenuity Pathway Analysis software.

| **ID** | **Molecules in Network** | **Score** | **No.**  **Focus**  **genes** | **Functions** |
| --- | --- | --- | --- | --- |
| **1** | **ADAMTS9,BGN,C3AR1,CARD6,Cbp,COL1A1,COL3A1,COL6A1,COL6A2,COL6A3,COLEC12,collagen,Collagen type I,CTSK,CTSS,ETS,EXOC1,Inflammasome (Nalp1, Asc, Casp1, Casp4),ITPKB,KLF3,LY96,MEOX1,MEOX2,MRC2,NCOA7,NFkB (complex),NLRP1,PELO,PLK2,PLOD2,PTPLAD1,RFTN1,SERPINH1,TFPI2,TRAM2** | **43** | **29** | **Connective Tissue Disorders, Genetic Disorder, Skeletal and Muscular Disorders** |
| **2** | **Alpha Actinin,Alpha catenin,ANGPTL1,ANXA2,Cadherin,CAP1,CDH11,CLDN5,CTNNA1,CTNND1,ERK1/2,F Actin,F11R,F2R,FAM84B (includes EG:157638),G-Actin,IQGAP1,Itgam-Itgb2,JAM,JAM2,JAM3,KIRREL,MARCKSL1,MLLT4,MPZL1,NTN4,PROCR,PTPRB,RAB13,SDCBP,SNX9 (includes EG:51429),SPTBN1,TEK,TJP1,Tropomyosin** | **36** | **26** | **Cell-To-Cell Signaling and Interaction, Tissue Development, Cardiovascular System Development and Function** |
| **3** | **A2M,Actin,Alpha tubulin,Bcl9-Cbp/p300-Ctnnb1-Lef/Tcf,CD44,CFL1,Cofilin,DPYSL2,FN1,FZD6,GJA1,GLS,HDGFRP3,HIP1R,HTRA1,LIPA,LRP,Notch,NOTCH4,PARVA,PCDHGC3,PFDN2,Pkc(s),PTPN12,PTPRK,RAB8B,S100A11,Secretase gamma,SNTA1,SOX4,TCF4,TCF/LEF,TCF7L2 (includes EG:6934),TMOD3,UTRN** | **36** | **26** | **Cell Morphology, Cellular Development, Carbohydrate Metabolism** |
| **4** | **ACTG1,AIF1,APP,C1q,C1QA,C1QC,C1S,CALD1,CD93,CFH,CLIC1,CPE,DBN1,EMCN,EPB41L2,EPB41L3,ERG,ERP44,Fascin,FOXP1,ICAM2,Iga,Igm,Integrin alpha 5 beta 1,ITM2B,LDB2,Ldh,MARCKS,Mucin,MYH9,Na-k-atpase,PCSK5,PGAM1,SPARCL1,TSPAN12** | **35** | **27** | **Endocrine System Development and Function, Small Molecule Biochemistry, Cardiovascular Disease** |
| **5** | **AHR,ANKRD29,BTG3,CD164,CHSY1,CREB3L2,Cyclin A,Cyclin E,DDX5,DRAM1,E2f,EFHC1,ELOVL1,EML4,GADD45GIP1,Insulin,KLHDC5,KPNA2,MIR1,MIR124,MTMR12,PEA15,PP1 protein complex group,PTBP1,Ptk,Rab5,Rb,RBMS1,RCN3,SLC44A1,STEAP4,TAGLN2,TMSB4X,TWF1,ZC3HAV1** | **34** | **25** | **Infection Mechanism, Cell Cycle, DNA Replication, Recombination, and Repair** |
| **6** | **14-3-3,AIM1 (includes EG:202),Ap1,BCR,C14ORF153,CASP2,CASP4,CASP6,Caspase,CBL,CCDC59,CLIC4,CLPX,CSF1R,Cytochrome c,FGL2,GRN,Hsp27,Hsp90,LYN,MAP3K1,Mek,MITF,MTCH1,NGFRAP1,PDCD6IP,peptidase,PKD2 (includes EG:5311),PLAU,RAI14,TIMP1,TSHZ3,TUBB,Tubulin,YWHAQ (includes EG:10971)** | **33** | **25** | **Cell Death, Cell-To-Cell Signaling and Interaction, Embryonic Development** |
| **7** | **ARHGDIB,ASPN,COL1A2,DACH1,ETS1,FLI1,FMR1,GUCY1B3,IFN ALPHA RECEPTOR,KDR,KIT,LASP1,LOX,MYOF,Nuclear factor 1,Pdgf,PECAM1,PI3K,RNASE1,RNase A,S1PR1,Sfk,SH2B3,SIX1,Smad,Sphk,SPRED1,SRC,SRGN,STAT5a/b,SWAP70,TGFBR2,TYROBP,Vegf Receptor,YES1** | **32** | **24** | **Cancer, Tissue Morphology, Cardiovascular System Development and Function** |
| **8** | **Akt,ANTXR1,COL15A1,COL18A1,COL4A1,COL4A2,Collagen type III,Collagen type IV,DAPK1,FBLN2,Fibrin,Flotillin,Igf,IGF2,Igfbp,IGFBP7,LAMA4,LAMB1,LAMC1,Laminin,Laminin1,Laminin2,LGALS3,LGALS3BP,Mmp,MMP2,NID1,NID2,OGN,PCOLCE,PPT1,PROS1,SNAI2,TACC1,THBS4** | **30** | **24** | **Connective Tissue Disorders, Genetic Disorder, Cardiovascular System Development and Function** |
| **9** | **Adaptor protein 2,ADCY7,AKAP12,Angiotensin II receptor type 1,Arf,ARF3,ASAP1,Beta Tubulin,Clathrin,CLTC,COPB1,COPB2,DAB2,ERK,FSCN1,FSH,Ifnar,IL6ST,JAK,Lh,LOXL1,P4HA2,PICALM,PTPRE,RAB31,RAB1A,RAB5C,RASSF2,RECK,SOCS2,STAT,TNFAIP6,TPM4,TUBA1A,tyrosine kinase** | **30** | **23** | **Genetic Disorder, Ophthalmic Disease, Carbohydrate Metabolism** |
| **10** | **ARHGAP1,ARHGAP29,C3-Cfb,CBX4,CD46,CD81,CFI,Ctbp,DLC1,Erm,Fc receptor,Jnk,KLHL2,LGALS8,MCF2L,MECOM,Mlc,MSN,MYH10,MYL6,Myosin,Pde,PDE1A,PDE8A,PTGFRN,RAD50,Ras homolog,RBBP8,RHOG,RhoGap,RHOJ,Rock,SMC1A,STK17B,THY1** | **28** | **24** | **Cell Signaling, Cell-To-Cell Signaling and Interaction, Hematological System Development and Function** |
| **11** | **ABI1,ACTR2,ACTR3,Alpha actin,Arp2/3,ARPC5,BCL10,CD3,CYFIP1,EPS8,Fcer1,Fcer1a-Fcer1g-Ms4a2,FCGR1A/2A/3A,FSTL1,FYN,HCLS1,HHEX,MRC1,NCK,NFAT (complex),NRP1,P38 MAPK,PAG1,Pak,Pdgfr,POSTN,PTPRC,Rac,RGS5,Sos,SYK/ZAP,TRAF5,VAV,WIPF1,WNK2** | **24** | **20** | **Cell-To-Cell Signaling and Interaction, Cellular Assembly and Organization, Cell Morphology** |
| **12** | **ATP1B3,B2M,CANX,DEGS1,EMP1,FABP5,FCER1G,Fcgr2,FCGR2B,FKBP11,FKBP1A,IFN Beta,Ige,IgG,IgG2a,IL12 (complex),Immunoglobulin,KCTD12,KDM5B,Mediator,MHC Class I (complex),MHC CLASS I (family),MHC Class II (complex),MICAL2,peptidylprolyl isomerase,PLC gamma,PLS3,PPIA (includes EG:5478),PPIB,PTMA,RNASET2,RRBP1,SAA,STAB1,TCR** | **24** | **20** | **Drug Metabolism, Small Molecule Biochemistry, DNA Replication, Recombination, and Repair** |
| **13** | **AIF1,AIM2,alcohol group acceptor phosphotransferase,CD33 (includes EG:945),CYB561,DAPK1,DRG1,GGTA1,GRK4,Hla-abc,IFI44,IFITM2,IFITM3,IFNA2,IFNG,inosine,IRG1,KCTD20,KLRA17,LCP1,LGALS3BP,LPAR6,MAP3K8,MS4A6A,NPL,PARVG,PHF11 (includes EG:51131),PRKCH,progesterone,ROD1,SAMD9,TRIL,TRIM22,TTC28,TTK** | **24** | **20** | **Antimicrobial Response, Inflammatory Response, Infection Mechanism** |
| **14** | **20s proteasome,Calpain,Caspase 3/7,CAV1,CAV2,Caveolin,CD53,CDK6,Collagen Alpha1,Collagen(s),Eotaxin,FBN1,FERMT2,Fibrinogen,Filamin,Focal adhesion kinase,IGF1,Integrin,Integrin alpha 3 beta 1,Integrinα,Integrinβ,ITGB1,MAP2K1/2,MOBKL1B,NOSIP,PP2A,SHC1,SPARC,STK38,STK38L (includes EG:23012),STOM,TM4SF1,TRIP6,UBE2B,VCAN** | **23** | **19** | **Cell Morphology, Connective Tissue Development and Function, Cell-To-Cell Signaling and Interaction** |
| **15** | **ABI3BP,ADD3,ATP5D,ATP5G1,ATP6V1B2,B3GNT2,CSRP2BP,DCAF6,DDB1,DOCK9,FAM101B,FGF1,HIST1H4C,HTT,KAT2A,LOXL2,LRRC17,MLF2,MPEG1,PHLDB2,POSTN,RAI14,retinoic acid,SASH1,SEPHS1,SFXN3,SLC25A12,SOX7,SOX17,SPTBN2,TACC1,TMSB10,TPR,YEATS2,YWHAZ** | **23** | **19** | **Cellular Compromise, Skeletal and Muscular System Development and Function, Cell Cycle** |
| **16** | **CASP1,CCL2,CD58,CD163,CHEMOKINE,IFI16,IFI27,IFI44,IFITM2,IFITM3,Ifn,IFN alpha/beta,Ifn gamma,IFN TYPE 1,IFNAR1,IFNGR1,IKK (complex),Ikk (family),IL1,IL-1R,IL12 (family),IL1R1,Interferon alpha,IRF8,IRG,LYZ,MYD88,PARP,PARP4,Tlr,TLR2/3/4/9,Tnf,TNFSF10,TNFSF13B,XAF1** | **22** | **19** | **Connective Tissue Disorders, Inflammatory Disease, Skeletal and Muscular Disorders** |
| **17** | **ACLY,ADD3,ALOX5,ANXA1,ANXA5,Calcineurin protein(s),Calmodulin,CaMKII,CAPN2,CAPN6,Ck2,COX7C (includes EG:1350),Creb,CREB5,Cytochrome c oxidase,EBAG9,FGFR1,Gsk3,Histone H1,Histone h3,HOXA9,KL,LPHN2,MAGED2,MID1,MYO1B,Nfat (family),NMDA Receptor,Pka,PPP1R16B,PTEN,RNA polymerase II,TM4SF18,TMSB10,Vegf** | **21** | **21** | **Cell-To-Cell Signaling and Interaction, Hematological System Development and Function, Immune Cell Trafficking** |

The first column represent the ID of the network, the second column shows the molecules in the network. The third column is the score of the network. The forth column shows the number of the genes in a network. The final and the fifth column shows the top functions describing the network.

**Table S8. MYOD targets which are significantly different in response to training in healthy patients (FDR 10%, fold> 1.5).**

| Genes | CS/CT | FDR | NS/NT | FDR | WS/WT |
| --- | --- | --- | --- | --- | --- |
| MYO1B | 2.424319 | 0.003434 | 2.358684 | 0.043488 | NS |
| MYH10 | 1.9779 | 0.010815 | NS | NS | NS |
| MYH9 | 1.769668 | 0.01157 | NS | NS | NS |
| MYO15B | 1.680785 | 0.053249 | NS | NS | NS |
| MYLK2 | -1.73438 | 0.060255 | NS | NS | NS |
| MYO6 | 1.680994 | 0.060255 | NS | NS | NS |

None of the MYOD targets were significantly different at this threshold.

**Table S9. Known Validated Targets of NFKB**

**Table S10. Functional annotation of the significantly different genes identified by a two factor ANOVA. The factors are exercise training and disease status.**

| **Functional category** | **#of genes** | **Factor** |
| --- | --- | --- |
| GOBP RNA processing | 121 | Disease |
| GOBP ribonucleoprotein complex biogeneis | 53 | Disease |
| GOCC mitochondrion | 198 | Disease |
| GOCC contractile fiber | 33 | Disease |
| KEGG Tight junction | 35 | Disease |
| GOCC Chromatin remodelling complex | 19 | Disease |
| GOCC histone deacytylase complex | 12 | Disease |
| GOMF RNA binding | 245 | Exercise |
| GOBP proteolysis involved in cellular protein catabolic process | 206 | Exercise |
| GOMF enzyme binding | 182 | Exercise |
| GOCC ribonucleoprotein complex | 165 | Exercise |
| GOBP protein complex biogenesis | 166 | Exercise |
| GOCC extracellular matrix part | 50 | Exercise |
| GOBP blood vessel development | 89 | Exercise |
| GOBP regulation of cell proliferation | 236 | Exercise |
| GOCC contractile fiber | 50 | Exercise |
| KEGG ECM-receptor interaction | 36 | Exercise |
| GOCC histone deacetylase complex | 17 | Exercise |
| GOCC ribonucleoprotein complex | 29 | Interaction |
| GOMF GTPase activity | 14 | Interaction |
| GOBP RNA processing | 21 | Interaction |
| GOCC mitochondrial part | 20 | Interaction |

The functions noted by * are functionally enriched (FDR < 10%).
